# The crosstalk between breast carcinoma-associated fibroblasts and cancer cells promotes RhoA-dependent invasion *via* IGF-1 and PAI-1

**DOI:** 10.18632/oncotarget.23735

**Published:** 2017-12-28

**Authors:** Julien Daubriac, Shiwei Han, Jelena Grahovac, Eve Smith, Abdel Hosein, Marguerite Buchanan, Mark Basik, Yves Boucher

**Affiliations:** ^1^ Edwin L. Steele Laboratory for Tumor Biology, Massachusetts General Hospital, Boston, Massachusetts, USA; ^2^ Lady Davis Institute for Medical Research, Sir Mortimer B. Davis Jewish General Hospital, McGill University, Montreal, Canada

**Keywords:** carcinoma-associated fibroblasts, RhoA/ROCK signaling, insulin-like growth factor-1, plasminogen activator inhibitor-1, cancer cell scattering and invasion

## Abstract

Carcinoma-associated fibroblasts (CAFs) can remodel the extracellular matrix to promote cancer cell invasion, but the paracrine signaling between CAFs and cancer cells that regulates tumor cell migration remains to be identified. To determine how the interaction between CAFs and cancer cells modulates the invasiveness of cancer cells, we developed a 3-dimensional co-culture model composed of breast cancer (BC) MDA-MB-231 cell spheroids embedded in a collagen gel with and without CAFs. We found that the crosstalk between CAFs and cancer cells promotes invasion by stimulating the scattering of MDA-MB-231 cells, which was dependent on RhoA/ROCK/phospho MLC signaling in cancer cells but independent of RhoA in CAFs. The activation of RhoA/ROCK in cancer cells activates MLC and increases migration, while the genetic-down-regulation of RhoA and pharmacological inhibition of ROCK reduced cell scattering and invasion. Two distinct mechanisms induced the activation of the RhoA/ROCK pathway in MDA-MB-231 cells, the secretion of IGF-1 by CAFs and the upregulation of PAI-1 in cancer cells. In an orthotopic model of BC, IGF-1R inhibition decreased the incidence of lung metastasis, while Y27632-inhibition of ROCK enhanced the lung metastasis burden, which was associated with an increased recruitment of CAFs and expression of PAI-1. Thus the crosstalk between CAFs and BC cells increases the secretion of IGF-1 in CAFs and PAI-1 activity in cancer cells. Both IGF1 and PAI-1 activate RhoA/ROCK signaling in cancer cells, which increases cell scattering and invasion.

## INTRODUCTION

In triple-negative breast cancer (TNBC) and other breast cancer subtypes, the presence of carcinoma-associated fibroblasts (CAFs) and collagen fibers correlates with high-grade malignancies and reduced survival [1]. CAFs have been shown to participate in tumor progression by enhancing invasion, metastasis, cancer cell survival and drug resistance [2, 3]. This multiplicity of effects has to be put in perspective with the heterogeneity of the CAF population, as they have been shown to originate from resident fibroblasts, adipocytes, epithelial and endothelial cells as well as from bone marrow-derived mesenchymal and hematopoietic stem cells [3–5]. In comparison to normal stromal cells, CAFs display multiple molecular alterations that promote their proliferation and survival and modulate the secretion of cytokines and extracellular matrix (ECM) components [6, 7]. In response to molecular signals from cancer cells (e.g. TGF-β, SDF-1) CAFs adopt an activated myofibroblast phenotype, characterized notably by an increased expression of α-smooth muscle actin (α-SMA) and synthesis of collagen [8]. This desmoplastic reaction modulates the adhesion of cancer cells, leading to the remodeling of a permissive and supportive environment for tumor progression and dissemination [9–12].

Disseminating cancer cells undergo several molecular and cellular changes that modulate their intercellular and matrix adhesions as well as their mode of migration [13]. The loss of E-cadherin and the activation of epithelial-mesenchymal transition (EMT) are considered hallmarks of metastasis in several human cancers. In basal-like breast cancer the low expression of E-cadherin correlates with poor clinical outcome [14]. The loss of E-cadherin not only alters tissue homeostasis, allowing cancer cells to dissociate from the primary mass, but also promotes migration by mediating the activity of receptor tyrosine kinases [15]. CAFs have been shown to down-regulate the expression of E-cadherin and increase the migration velocity of breast cancer cells [16, 17].

Cancer cells display at least two distinct modes of single-cell migration, which are regulated by Rho family proteins (including RhoA, Rac1 and Cdc42) [18]. RhoA and its downstream effector ROCK mediate a proteolysis-independent amoeboid movement (rounded displacement) while Rac1 and Cdc42 drive the cells toward a mesenchymal type of migration (elongated displacement) [19–21]. The RhoA-dependent amoeboid movement is characterized by low-adhesion to matrix substrates and higher migration speeds, while Rac1-dependent mesenchymal movement involves adhesion and proteolytical degradation of the ECM and is generally associated with lower migration speeds [19]. The amoeboid movement of cancer cells is a significant mode of migration in collagen gels in the absence of efficient pericellular proteolysis [22]. The disruption of the activity of RhoA or Rac1/Cdc42 is associated with malignancy [20, 23], and in invasive breast ductal carcinoma, the overexpression of either RhoA or Rac1 have been associated with worse prognosis [24]. In addition, ROCK1 and ROCK2 expression is increased in breast cancer specimens from patients with nodal metastasis [25, 26].

The architecture and composition of the ECM affect the mode of migration of cancer cells [22, 27, 28]. CAFs have been shown to remodel the ECM by creating tracks, which facilitate the collective migration of squamous carcinoma cells [29, 30]. The contractile forces that mediate matrix fiber alignment and stiffness are notably dependent on the expression of caveolin-1 and p190RhoGAP [11]. However, how the paracrine signaling between CAFs and cancer cells directly modulate the migration phenotype of cancer cells has not been studied in detail. To study how the interaction between CAFs (isolated from human invasive breast cancer including TNBC) and TNBC cells affects TNBC cell scattering and motility, intercellular contacts and cellular signaling, we developed a three-dimensional (3D) co-culture model of cancer cell spheroids and CAFs embedded in a collagen gel. Our results show that the crosstalk between cancer cells and CAFs promotes cellular scattering and invasion. The crosstalk between CAFs and cancer cells activates RhoA/ROCK signaling in cancer cells, which is dependent on the secretion of IGF-1 by CAFs and PAI-1 upregulation in cancer cells. We also investigated the significance of this crosstalk in a metastatic breast cancer model in mice.

## RESULTS

### The crosstalk between CAFs and MDA-MB-231 cells promotes invasion and scattering

**Figure 1:**
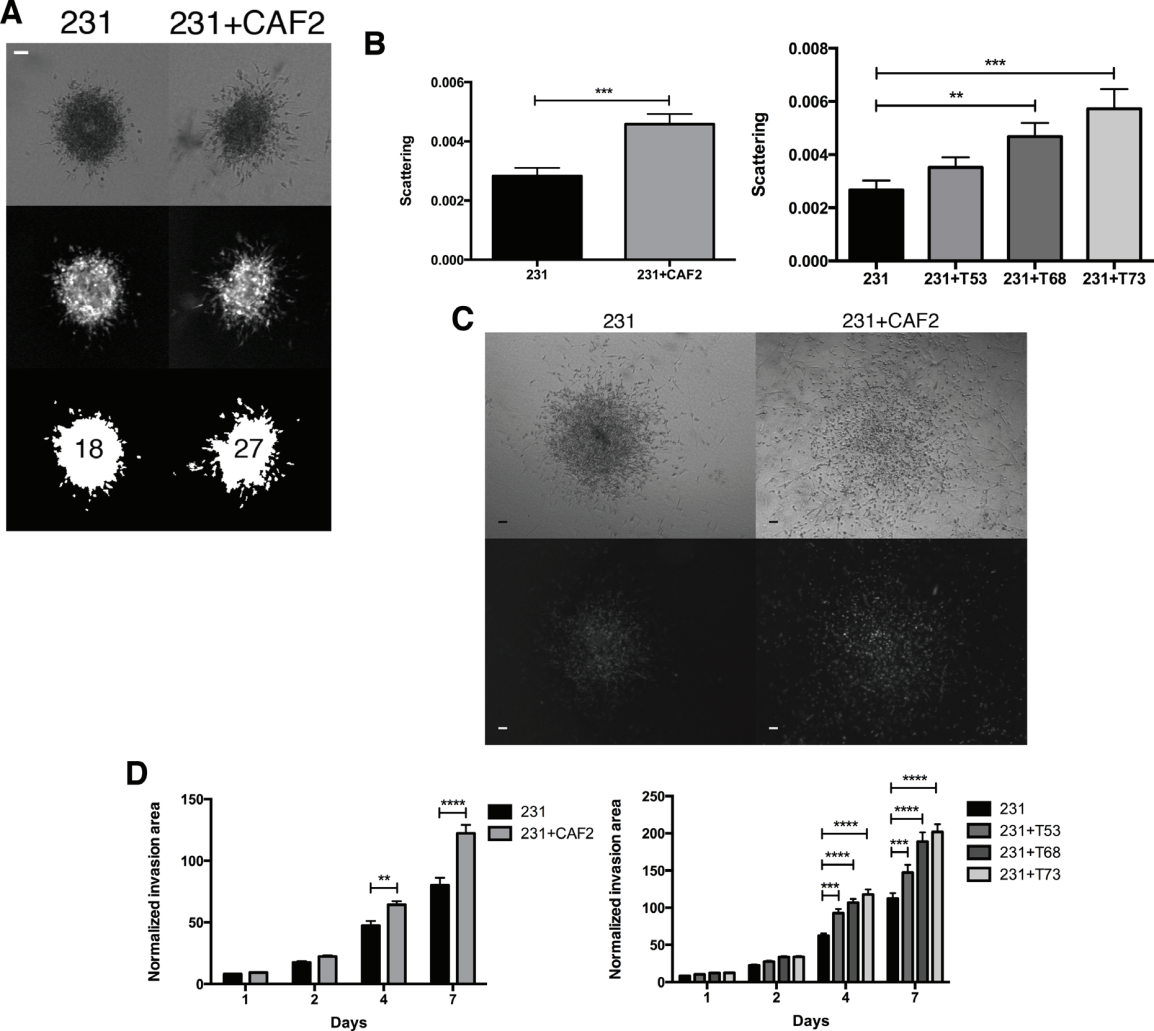
CAFs increase the scattering and invasion of MDA-MB-231 cancer cells MDA-MB-231 spheroids cultured without or with CAF2 in a collagen gel over a 7-day period. (**A–B**) At day 1, scattering was evaluated by counting the number of GFP^+^ single MDA-MB-231 cells surrounding the spheroid. (**C**) Kinetic of GFP^+^ MDA-MB-231 cells invasion with or without CAFs in collagen gels: The normalized invasion area was defined as the GFP ratio of the invasion area to the size of the spheroid at day 0. Brightfield (top) and fluorescent (bottom) images showing spheroids and the invasion of 231 cells at distance from center of spheroids. (**D**) On day 4 and 7 CAFs significantly increased the invasion of MDA-MB-231 cells. Data expressed as mean ± SEM. ^*^p ≤ 0.05, ^**^p ≤ 0.01, ^***^p ≤ 0.001, ^****^p ≤ 0.0001. Bar 100 μm.

The CAFs used for our experiments were isolated from a human invasive breast cancer of unknown subtype (CAF2; [31]), while the other CAF cell lines were isolated from human TNBC lesions [32] (see methods for additional information). To determine whether CAFs increase the invasion of BC cells, we incorporated MDA-MB-231 breast cancer spheroids into a collagen gel with or without CAFs and measured cell scattering and the area of cancer cell invasion. Within 24 h CAF2 and 2 of 3 CAF TNBC cell lines induced a significant scattering of cancer cells, as determined by an increase in the number of single cells surrounding the spheroids (Figure 1A and 1B). On days 4 and 7, CAF2 and 3 of 3 TNBC CAF cell lines significantly increased the invasion of MDA-MB-231 cells (Figure 1C and 1D). Cells that invaded the surrounding collagen gel had elongated or rounded shapes (Supplementary Figure 1A). There was no difference in cell aspect ratio (major axis / minor axis = ~2) or circularity (Supplementary Figure 1A) between spheroids cultured with or without CAFs.

### The crosstalk between CAFs and BC cells induce the activation of the RhoA/ROCK pathway in MDA-MB-231 cells

**Figure 2:**
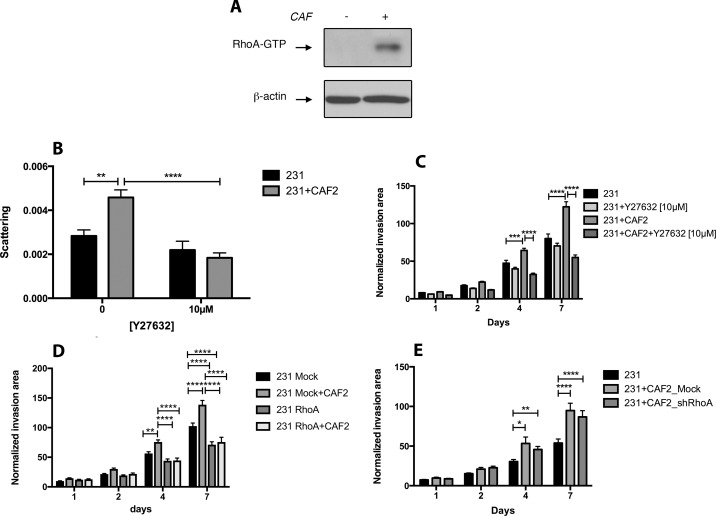
CAFs promote MDA-MB-231 invasion and scattering by activating RhoA/ROCK in cancer cells (**A**) Effect of CAFs on RhoA-GTP expression in MDA-MB-231 cells: MDA-MB-231 spheroids were culture with or without CAF2 for 72 h and assayed for RhoA activation by RhoA-GTP pulldown assay. β-actin was used as a loading control. (**B–C**) Effect of Y27632 [10 μM] on the scattering and invasion of MDA-MB-231 cells cultured with or without CAF2 in a collagen gel. (**D**) Kinetic of RhoA-silenced MDA-MB-231 cells invasion with or without CAFs in collagen gel. (**E**) Kinetic of GFP^+^ MDA-MB-231 cells invasion with RhoA-silenced or mock-transfected CAFs in collagen gel. Data expressed as mean ± SEM. ^*^p ≤ 0.05, ^**^p ≤ 0.01, ^***^p ≤ 0.001, ^****^p ≤ 0.0001.

Because the movement of rounded cells is linked to the activation of the RhoA/ROCK pathway, we determined whether CAFs could activate this pathway in MDA-MB-231 cells. We found that the co-culture of CAF2 and MDA-MB-231 cells increased the expression of RhoA-GTP in MDA-MB-231 cells (Figure 2A). We then assessed the effect of the ROCK inhibitor Y27632 on BC cell scattering and invasion. Y27632 did not affect the scattering and invasion of MDA-MB-231 cultured without CAFs, but significantly reduced the scattering (Figure 2B) and invasion induced by CAFs (Figure 2C, Supplementary Figure 1B and 1C), suggesting that CAFs promote the invasion of MDA-MB-231 cells *via* ROCK1/2. To confirm that CAFs promote cancer cell invasion by activating RhoA in MDA-MB-231 cells, we used shRNA interference to reduce the expression of RhoA in both cancer cells and CAFs (Supplementary Figure 1D and 1E). In RhoA-silenced cancer cell spheroids, CAFs did not increase invasion, confirming the role of RhoA-activation in promoting CAF-induced invasion (Figure 2D). RhoA silencing also reduced the invasion of MDA-MB-231 cells without CAFs, hence part of the invasion of MDA-MB-231 cells is also dependent on the activity of RhoA. Because RhoA-dependent remodeling / contraction of the ECM by CAFs can promote cancer cell migration we also used shRNA constructs to knockdown the expression of RhoA in CAFs. The silencing of RhoA in CAFs reduced the expression of α-SMA and MMP14 (Supplementary Figure 1E), but did not reduce the effects of CAFs on MDA-MB-231 cell invasion (Figure 2E). These findings suggest than in our 3D co-culture model, CAFs promote MDA-MB-231 invasion through secreted soluble factors rather than through a force-dependent remodeling of the ECM.

### TNBC cells increase the secretion of IGF-1 in CAFs

**Figure 3:**
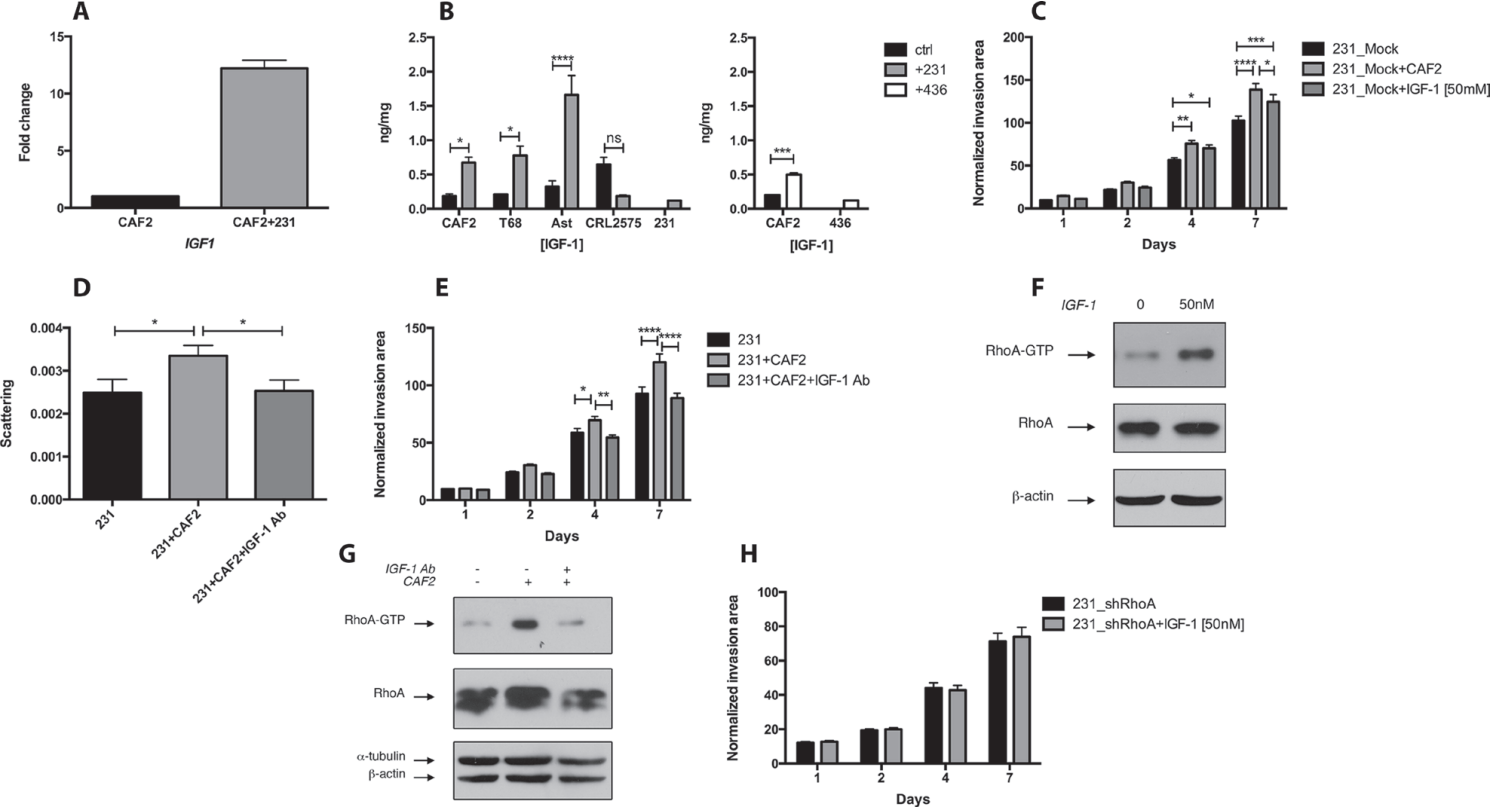
CAFs promote RhoA/ROCK-dependent invasion and scattering of MDA-MB-231 cells *via* IGF-1 (**A**) CAF2 were cultured with or without MDA-MB-231 cells for 72 h and assayed for *IGF1* expression by RT-PCR. MDA-MB-231 cells increase *IGF1* in CAF2 by 12 fold. (**B**) Similarly, several CAFs and CRL2575 cells were cultured with or without MDA-MB-231 or MDA-MB-436 cells for 72 h in serum-free condition, and supernatants were assayed for IGF-1 expression by ELISA. MDA-MB-231 increased IGF-1 secretion in all CAFs tested but not in CRL2575. MDA-MB-436 cells increased IGF-1 secretion in CAF2. (**C**) Effect of recombinant IGF-1 [50 mM] on collagen invasion of MDA-MB-231. (**D–E**) Effect of IGF-1 blocking antibody [20 μg/ml] on scattering and invasion of MDA-MB-231 cells cultured with or without CAF2 in collagen gel. (**F**) Effect of recombinant IGF-1 on RhoA-GTP expression in MDA-MB-231 cells: MDA-MB-231 spheroids were culture with or without [50 nM] recombinant IGF-1 for 15 min and assayed for RhoA activation by RhoA-GTP pulldown assay. (**G**) Effect of IGF-1 blocking antibody on RhoA-GTP expression in MDA-MB-231 cells: MDA-MB-231 spheroids were culture with or without CAF2 and treated with an IGF-1 blocking antibody for 72 h and assayed for RhoA activation. (**H**) Effect of recombinant IGF-1 on collagen invasion of RhoA-silenced MDA-MB-231 cells. Data expressed as mean ± SEM. ^*^p ≤ 0.05, ^**^p ≤ 0.01, ^***^p ≤ 0.001, ^****^p ≤ 0.0001.

In order to establish whether secreted factors could be responsible for CAF-promoted invasion, we measured by RT-qPCR array the transcription level of several genes related to EMT between CAFs and CAFs co-cultured with TNBC cells. In a transwell co-culture system (where CAFs and cancer cells were physically separated), MDA-MB-231 cells increased the transcription of *IGF1* by 12 fold in CAFs (Figure 3A). In MDA-MB-231 cells alone or co-cultured with CAFs, *IGF1* could not be detected (Ct > 35) (Supplementary Figure 2A). The expression of *DPP4* and *ITGB3* in CAFs were both increased by 2.5 fold, while the expression of *EGF*, *FGF2*, *HGF* or *TGFB1* was not affected by MDA-MB-231 cells (Supplementary Figure 2B). Next, we measured by ELISA the secretion of IGF-1 in the supernatant of CAFs alone or co-cultured with cancer cells. MDA-MB-231 cells significantly increased the secretion of IGF-1 in all CAFs tested, but did not affect the expression of IGF-1 in a normal fibroblast cell line (Figure 3B). The TNBC cell line MDA-MB-436 also increased the secretion of IGF-1 in CAF2 (Figure 3B).

### IGF-1 increases RhoA signaling and invasion in MDA-MB-231 cells

We then tested the effect of recombinant IGF-1 on cancer cell invasion. IGF-1 [50 nM] significantly increased the invasion of MDA-MB-231 cells (Figure 3C). Reciprocally, antibody blockade of IGF-1 significantly reduced the CAF-enhanced scattering and invasion of MDA-MB-231 cells (Figure 3D and 3E). To determine whether IGF-1 promotes cancer cell invasion *via* the RhoA/ROCK pathway, we examined the effect of recombinant IGF-1 on RhoA-GTP expression. IGF-1 [50 nM] activated RhoA within 15 min (Figure 3F) and the IGF-1 blocking antibody reduced CAF-induced RhoA-GTP expression in MDA-MB-231 cells (Figure 3G). Finally, we tested the effect of recombinant IGF-1 on invasion in RhoA-silenced MDA-MB-231 cells. In comparison to mock-transfected cells, IGF-1 did not increase the invasion of RhoA-silenced cells (Figure 3C and 3H). Hence, our results show that TNBC cells increase the CAF-secretion of IGF-1, which enhances RhoA/ROCK-dependent invasion.

To assess the role of *adherens* junction proteins – in CAF-promoted scattering of tumor cells – we examined the expression of E-cadherin and phospho-p120 catenin, which regulates RhoA activity. In particular, the phosphorylation of Tyr228 increases the binding affinity of p120 catenin for RhoA, maintaining RhoA in an inactive (GDP) form [33]. Three of 3 CAFs and IGF-1 reduced the expression of E-cadherin and the phosphorylation of p120 catenin although to different extents (Supplementary Figure 2C and 2D). Interestingly, the addition of Y-27632 to either co-cultures of CAF2 and MDA-MB-231 or IGF-1-treated MDA-MB-231 cells increased the expression of E-cadherin and phospho-p120 catenin, which suggest that their downregulation is ROCK-dependent. However, immunofluorescence indicated that E-cadherin is mostly expressed in the cytoplasmic compartment in MDA-MB-231 spheroids suggesting that E-cadherin might not have a significant effect on cell-cell adhesion or in hindering the scattering or invasion of MDA-MB-231 cells in presence of CAFs (Supplementary Figure 2E).

### CAFs increase PAI-1 expression in MDA-MB-231 cells independently of IGF-1

**Figure 4:**
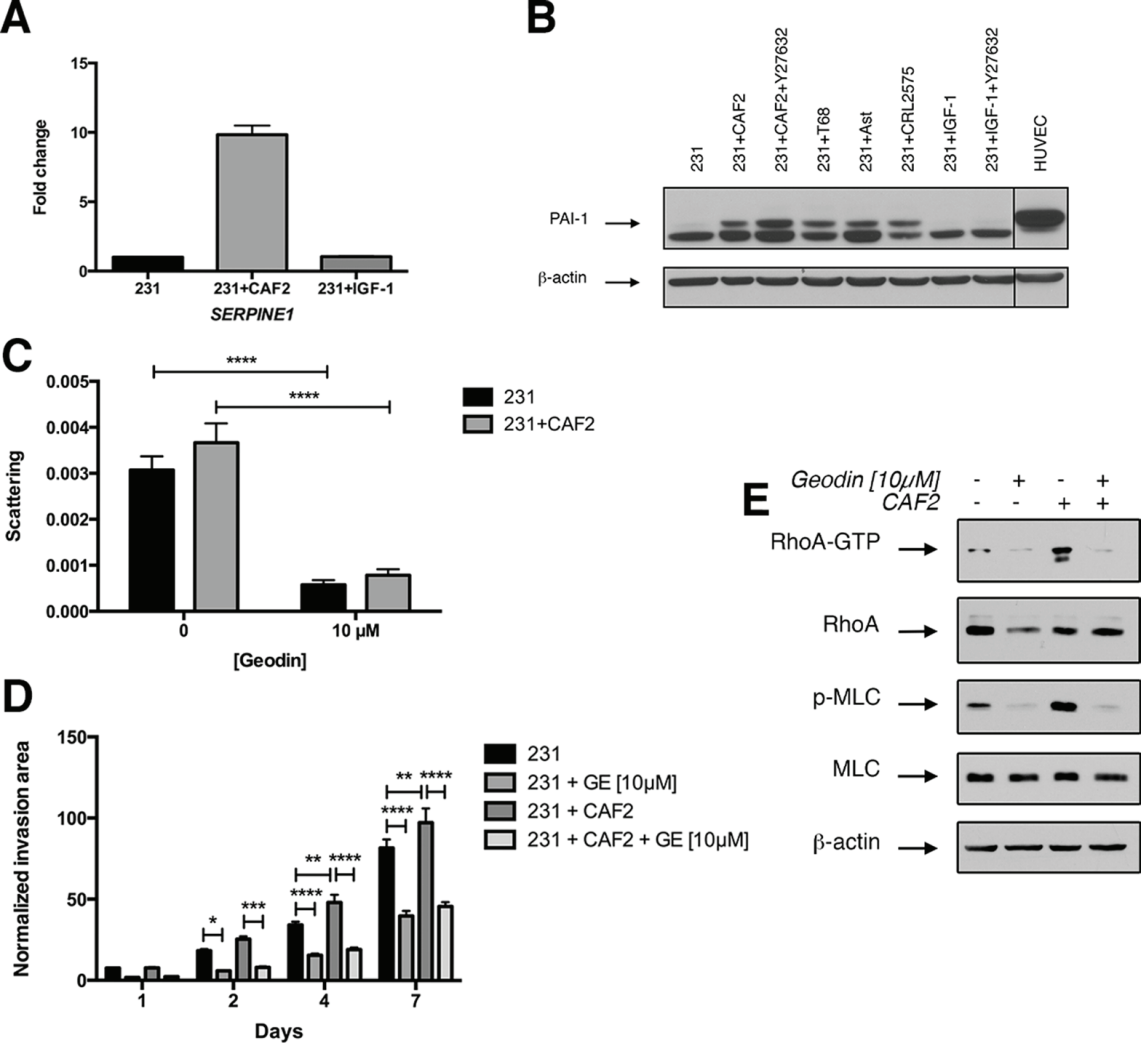
CAFs but not IGF-1 increase PAI-1 expression in cancer cells (**A**) MDA-MB-231 spheroids were culture alone or in presence of CAFs or IGF-1 [50 nM] for 72 h and assayed for *SERPINE1* (*PAI1*) expression by RT-PCR. (**B**) MDA-MB-231 cell spheroid were cultured alone or in presence of CAFs, IGF-1 [50 nM] and/or Y27632 [10 μM] for 72 h and immunoblotted for PAI-1 and β-actin as loading control. HUVECs were used as a positive control for PAI-1 expression. (**C–D**) Effect of PAI-1 inhibition on scattering and invasion of MDA-MB-231 cells in a collagen gel. MDA-MB-231 spheroids cultured with or without CAF2 in a collagen gel over a 7-day period in absence or in presence of 10 μM of geodin. (**E**) Effect of PAI-1 inhibition on RhoA pathway activation in MDA-MB-231 cells: MDA-MB-231 cells were cultured alone or in presence of CAF2 in absence or presence of 10 μM of geodin and assayed for RhoA activation by RhoA-GTP pulldown assay and immunobloted for phospho-MLC (Thr18/Ser19). Data expressed as mean ± SEM. ^*^p ≤ 0.05, ^**^p ≤ 0.01, ^***^p ≤ 0.001, ^****^p ≤ 0.0001.

To determine whether the crosstalk between cancer cells and CAFs modulate EMT, migration pathways in cancer cells we analyzed the expression of 84 EMT-related genes with a PCR array. The co-culture of CAF2 and MDA-MB-231 cells enhanced the transcription of *SERPINE1* (*PAI-1*) (Figure 4A). CAF2 also increased the expression of *KRT19* by 10-fold and *MMP3* by 6-fold in MDA-MB-231 cells (Supplementary Figure 3A). At the protein level, we confirmed that all CAFs and also normal fibroblasts increased levels of the full-length form of PAI-1 in MDA-MB-231 cells (Figure 4B). However, IGF-1 did not increase the expression of PAI-1 in MDA-MB-231 cells. Of note, MDA-MB-231 cells also express a lower molecular band of PAI-1 that could correspond to a truncated form of the protein.

Next, we tested the effect of geodin – a small molecule that inactivates PAI-1 – on cell scattering and invasion. At [10 μM], geodin had minimal effects on cell viability of CAFs and MDA-MB-231 cells (Supplementary Figure 3B–3C), but reduced the scattering and invasion of MDA-MB-231 cells, independently of the presence of CAFs (Figure 4C–4D, Supplementary Figure 3E), indicating that the basal expression of PAI-1 is required for invasion. In comparison to Y27632 inhibition of ROCK and antibody blockade of IGF-1, geodin produced a greater inhibition of cell scattering (Geodin 80%, Y27632 54%, IGF-1 24%). We also tested the effect of geodin on the highly invasive murine breast cancer cell line 4T1, which does not express PAI-1. Geodin did not affect the invasion of 4T1 cells (Supplementary Figure 3F).

To determine whether RhoA activation was dependent on PAI-1 expression, we analyzed the effect of geodin [10 μM] on the expression of RhoA-GTP and phospho-MLC (Thr18/Ser19). Geodin decreased the expression of RhoA-GTP and phospho-MLC in MDA-MB-231 cells cultured with or without CAFs, suggesting that RhoA-activation by PAI-1 is linked to the expression of PAI-1 (Figure 4E). Interestingly, PAI-1 expression increased in cells treated with Y27632, suggesting the existence of a regulatory loop between PAI-1 and ROCK (Figure 4B). Furthermore, the expression of PAI-1 was not affected by IGF-1, which indicates that CAFs activate RhoA in MDA-MB-231 cells *via* two distinct pathways, the secretion of IGF-1 by CAFs and the upregulation of PAI-1 in MDA-MB-231 cells.

### Targeting IGF-1R, but not ROCK, reduces the incidence of MDA-MB-231 lung metastasis without affecting tumor growth

**Figure 5:**
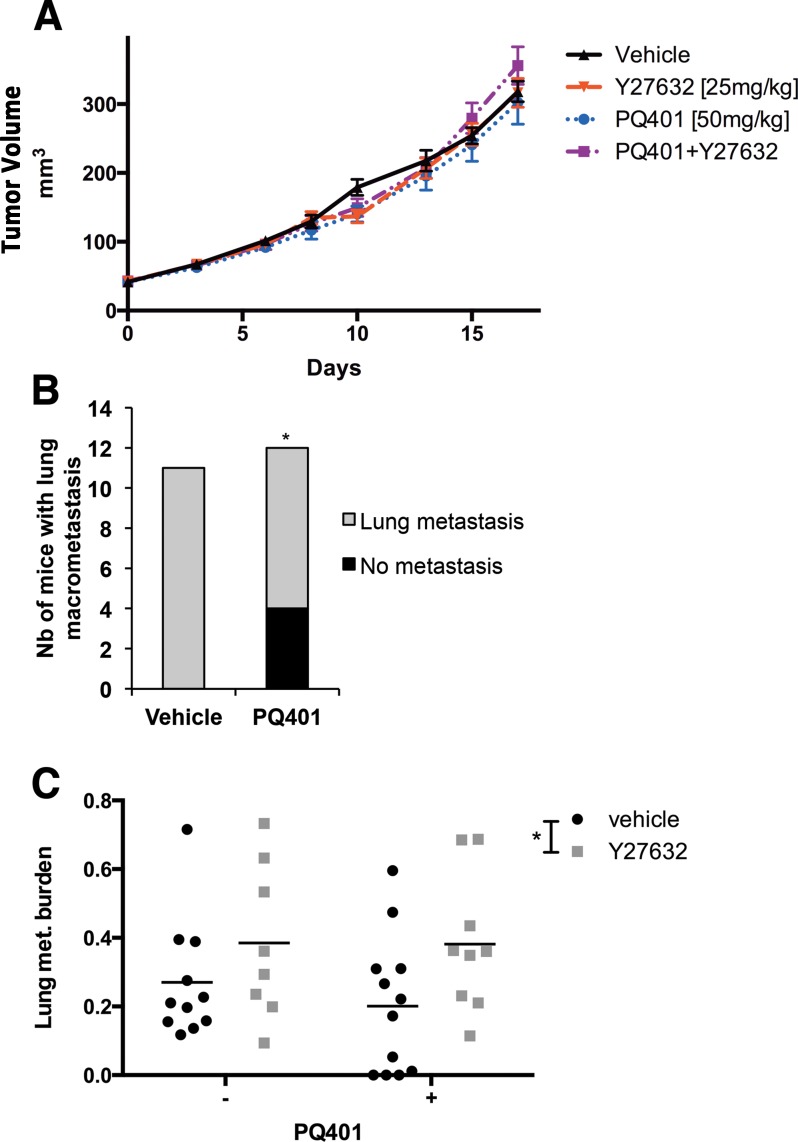
IGF-1R inhibition but not ROCK inhibition reduces the incidence of lung metastasis without affecting tumor growth in breast cancer xenografts (**A**) MDA-MB-231 cells were implanted in the mammary fat pad of SCID mice and treated with PQ401, Y27632 or PQ401 combined with Y27632. IGF-1R and ROCK inhibition did not affect primary tumor growth. (**B–C**) Five weeks after primary tumor resection, lungs of MDA-MB-231 tumor bearing mice were collected and analyzed for lung metastasis. PQ401 decreased non-significantly the metastasis burden in lungs, but significantly decreased the incidence of lung metastasis. Y27632 alone or combined with PQ401 significantly increased the lung metastasis burden.

In order to determine if targeting different components of the IGF-1/RhoA/ROCK crosstalk between BC cells and CAFs would affect primary tumor growth and metastasis, we treated MDA-MB-231 xenografts with the IGF-1R inhibitor PQ401 [34], Y27632 or PQ401 combined with Y27632. After 18 days of treatment, we did not observe any effect of PQ401, Y27632 or PQ401 combined with Y27632 on primary tumor growth (Figure 5A), suggesting that the inhibition of ROCK or IGF-1 receptor did not affect cancer cell proliferation. Five weeks after the resection of primary tumors, we analyzed the incidence of mice with metastasis and the metastasis burden in lung sections (fraction of lung tissue occupied by tumor). In the control group and mice treated with either Y27632 or Y27632 combined with PQ401, all mice showed evidence of macroscopic lung metastasis (Figure 5B). In contrast, in the PQ401-treated group the incidence of macroscopic lung metastasis was significantly less (*p* = 0.0351, Chi-squared test). Four of 12 mice had no macroscopic disease (3 mice had no lung metastasis and 1 mouse had microscopic disease) (Figure 5B). In comparison to the vehicle, PQ401-treated mice showed a 28% decrease in lung metastatic burden, the difference was not significant (*p* = 0.30, Two-way ANOVA), while Y27632 alone or combined with PQ401 significantly increased the metastatic burden (*p* = 0.0434, Two-way ANOVA) (Figure 5C).

### Inhibition of IGF-1 signaling and ROCK increases the expression of E-cadherin and phospho-p120 catenin in MDA-MB-231 tumors

To assess whether the inhibition of ROCK or IGF-1R was associated with change in *adherens* junctions as we observed *in vitro*, we analyzed the expression of E-cadherin and phospho-p120 catenin (Tyr228) by immunoblotting tumor lysates. We found that Y27632 significantly increased the expression of E-cadherin (*p* < 0.0001) and phospho-p120 catenin (*p* = 0.0071) (Supplementary Figure 4). In PQ401-treated tumors, there was a trend for an increased expression of E-cadherin (*p* = 0.0546) while only 3 tumors showed high phospho-p120 catenin expression. Moreover, when combined to PQ401, the effect of Y27632 on both E-cadherin, phospho-p120 catenin and PAI-1 expression was significantly diminished, indicating a possible interaction between the two drugs.

### Inhibition of ROCK increases the recruitment of CAFs and PAI-1 expression in MDA-MB-231 tumors

**Figure 6:**
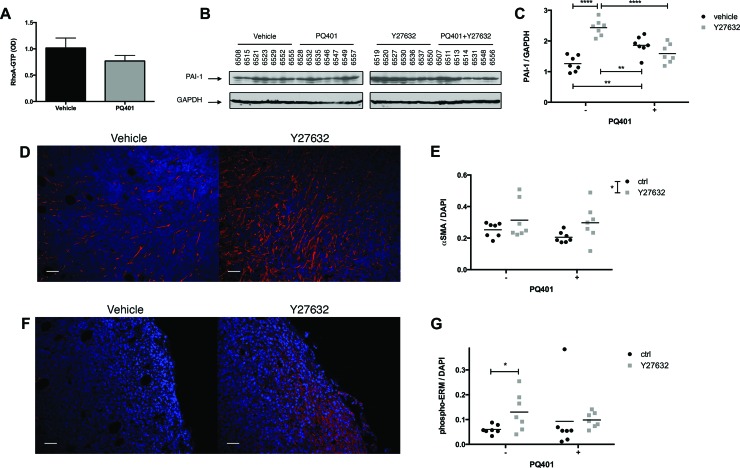
ROCK inhibition increases the recruitment of CAFs and PAI-1 expression in breast cancer xenografts (**A–C**) After 18 days of treatment, tumors were harvested and assayed for RhoA activation by G-LISA colorimetric assay (*n* = 6 per group), and for PAI-1 expression by immunoblotting. (**D**, **F**) Primary tumor sections were immunostained for α-SMA and phospho-ERM. (**E**, **G**) Y27632 or Y27632+PQ401 increases the number of α-SMA-positive cells in MDA-MB-231 primary tumors while only Y27632 significantly increases phospho-ERM expression. Bar 50 μm Data expressed as mean ± SEM. ^*^p ≤ 0.05, ^****^p ≤ 0.0001.

First, to validate the effect of IGF-1R inhibition on RhoA signaling, we quantified the level of RhoA-GTP expression in tumors treated with PQ401. We found that the PQ401-treated group showed a 24% decrease in RhoA-GTP level in comparison to the control group (ns) (Figure 6A). Then, to determine whether the level of PAI-1 was affected by the inhibition of ROCK in primary tumors, we analyzed its expression in tumor lysates. Similar to our *in vitro* findings, Y27632 significantly increased the expression of PAI-1, confirming the existence of a regulatory mechanism between ROCK and PAI-1 *in vivo* (Figure 6B and 6C). PQ401 induced a minor but significant increase in PAI-1 expression, while PQ401 combined with Y27632 did not affect PAI-1 expression. To better understand the difference in lung metastasis incidence between Y27632 and PQ401, we quantified the α-SMA expression in primary tumors by immunohistochemistry. In contrast to our *in vitro* data showing that RhoA down-regulation reduces α-SMA expression in CAFs, Y27632 significantly increased the number of α-SMA-positive cells, independently of the presence of PQ401, while PQ401 reduced the number of α-SMA-positive cells but not significantly (Figure 6D and 6E). Moreover, we found that the expression of the EMT marker phospho-ERM (Thr567/564/558) was increased by Y27632 but not by PQ401 (Figure 6F and 6G). Our results suggest that ROCK-inhibition in primary tumors promotes the recruitment of α-SMA-positive CAFs and expression of PAI-1, which may increase the invasiveness of cancer cells.

## DISCUSSION

Our findings show that in a 3D collagen gel the crosstalk between CAFs and BC cells promotes the invasion of MDA-MB-231 cells by stimulating RhoA GTPase, cell scattering and migration. In our model, this modulation of cancer cell invasiveness is not mediated by RhoA-dependent contractile forces of CAFs [11, 29, 30]. Instead the crosstalk between MDA-MB-231 cells and CAFs leads to increased migration, which depends on the activation of RhoA/ROCK/MLC signaling in cancer cells *via* two distinct mechanisms, the secretion of IGF-1 by CAFs and the upregulation of PAI-1 in cancer cells (Supplementary Figure 5).

IGF-1 signaling is known to induce EMT and promote invasion in BC cells [35]. The elevated expression of stromal IGF-1 and CXCL12 in primary TNBC lesions from patients has been linked with the selection of clones with high Src activity and bone metastasis formation [36]. Furthermore, the pharmacological – as shown in the present study – and genetic inhibition of IGF-1R reduces the incidence of lung metastasis [37]. Similar to our findings showing that BC cells increase the secretion of IGF-1 in CAFs, the tumor-conditioned media of TNBC cell lines – including MDA-MB-231 cells – stimulates the expression IGF-1 in human mesenchymal stem cells [36]. We show here that IGF-1 secretion by CAFs activates RhoA signaling in MDA-MB-231 cells, which promotes cell scattering and invasion. Interestingly, CAFs isolated from breast cancer lesions also secrete IGF binding proteins, which increases anoikis-resistance [38]. IGF-1R signaling also promotes the survival and apoptosis resistance [39]. Thus these findings suggest that the secretion of IGF-1 by CAFs supports the scattering, invasion and survival of BC cells during the initial steps of the metastatic cascade.

Independently of IGF-1, the crosstalk between CAFs and BC cells also increased the expression of PAI-1 in MDA-MB-231 cells, while geodin-inhibition of PAI-1 reduced RhoA activity in cancer cells, cancer cell scattering and invasion. Furthermore, geodin also reduced the scattering and migration of unstimulated MDA-MB-231 cell*s* – without CAFs –, which highlights the importance of PAI-1 in regulating the migration of MDA-MB-231 cells. PAI-1 can also promote the proliferation and survival of cancer cells [40], EMT [41], the migration and invasion of BC cells [42] and the amoeboid migration of colorectal cancer cells [43], as well as angiogenesis [44]. PAI-1 expression in cancer cells is regulated by TGF-β [45, 46]. CAFs secrete TGF-β to promote EMT in cancer cells [17]. Therefore, in parallel to IGF-1, TGF-β could contribute to the RhoA-dependent enhancement of cancer cell invasiveness *via* PAI-1.

The RhoA/ROCK pathway has been described in normal cells as a regulator of PAI-1 expression [47]. In contrast, our results show that geodin-inhibition of PAI-1 decreases RhoA activity in MDA-MB-231 cells and phospho-MLC (downstream target of RhoA) in HUVECs (Supplementary Figure 3G). Furthermore, we found that ROCK inhibition with Y27632, in parallel to the inhibition of its downstream target MLC (Supplementary Figure 6), increases both *in vitro* and *in vivo* the expression of PAI-1, suggesting the existence of a feedback loop between ROCK and PAI-1.

Although we identified a correlation between RhoA activation and tumor volume doubling time *in vivo* (Supplementary Figure 7), the Y27632 inhibition of ROCK did not affect primary tumor growth. Similar to our Y27632 findings, the knockout of either ROCK1 or ROCK2 in MDA-MB-231 cells did not affect tumor growth, whereas the intratumoral injection of anti-RhoA siRNA reduced tumor growth [48, 49]. Together, this suggests that the effect of RhoA on tumor cell proliferation could be independent of its downstream effectors ROCK1 and ROCK2. Another RhoA effector, mDia1, which works independently of ROCK, has been shown to contribute to cell cycle progression [50]. Contrary to our *in vitro* findings showing that the targeting of either RhoA or ROCK reduces CAF-enhanced invasion, the treatment of MDA-MB-231 xenografts with Y27632 increased lung metastasis. ROCK inhibition is known to facilitate the growth and survival of human embryonic stem cells by inhibiting anoikis. ROCK inhibitors are now part of standard stem cell culture protocols [51, 52]. Our results show that Y27632 can also increase the *in vitro* viability of MDA-MB-231 spheroids (Supplementary Figure 8). Therefore, it is possible Y27632 increased lung metastasis in MDA-MB-231 xenografts by promoting the survival of circulating tumor cells. We cannot either exclude that ROCK inhibition drove cancer cells toward a mesenchymal mode of migration [53]. In primary tumors, we found that Y27632 increased the recruitment of CAFs, PAI-1 expression and the phosphorylation of the pro-invasive proteins ERM (Ezrin, Radixin, Moesin), even if ROCK is a known ERM activator [54, 55]. In contrast, PQ401 did not affect the phosphorylation of ERM proteins and the number of α-SMA-positive cells. Although the blockade of ROCK reduces bone metastasis in MCF-7 xenografts overexpressing ROCK [26], our findings suggest that in primary MDA-MB-231 tumors Y27632 can also promote CAF recruitment and stimulate pro-invasive pathways, which may explain the increased lung metastasis formation.

In summary, we found that the crosstalk between CAFs and MDA-MB-231 BC cells increases the expression of IGF-1 in CAFs and PAI-1 activity in cancer cells. The increased expression of both IGF-1 and PAI-1 enhances RhoA signaling in cancer cells, which promotes cell scattering and invasion.

## MATERIALS AND METHODS

### Cell culture

Human mesenchymal-like breast cancer cell lines MDA-MB-231 (231) and MDA-MB-436 (436) were obtained from the American Type Culture Collection (ATCC). Cells were cultured in low glucose Dulbecco's modified Eagle's medium (DMEM) supplemented with 10% FBS in a humidified 5% CO2 incubator. Human breast CAF2 was obtained from Robert Weinberg [31]. The CAFs T53, T73 and T68 isolated from lesions of triple-negative breast cancer patients were characterized with antibodies as vimentin-positive and pan-cytokeratin-negative [32]. The Ast CAF (specimen 87322A1) – also isolated from triple-negative breast cancer lesions – were obtained from Asterand Inc. Normal human skin fibroblasts CRL-2575 were obtained from ATCC. All fibroblasts were cultured in DMEM supplemented with 10% FCS in a humidified 5% CO2 incubator. HUVECs were acquired from the Center for Excellence in Vascular Biology, Brigham & Women's Hospital, Harvard Medical School, Boston, MA and maintained in EGM medium (2% FBS, brain bovine extract, heparin, hEGF, and hydrocortisone) (Lonza). Cancer cell spheroids were generated by seeding 2 × 10^5^ cells/ml on low-adhesion surface (Corning) for 72 h. For RNA and protein extraction, indirect co-culture of cancer cell spheroids and CAFs was performed in 0.4 μm Transwell inserts (Corning).

### Antibodies and reagents

Y-27632 (Tocris), geodin (Santa-Cruz), blocking human IGF-1 antibody (R&D Systems, AF-291-NA) and recombinant IGF-1 (Sigma-Aldrich, I1146) were used in collagen invasion assay. The following antibodies: E-cadherin (Cell Signaling, #3195), phospho-p120 catenin (Tyr228) (Cell Signaling, #2911), PAI-1 (Cell Signaling, #11907), phospho-ERM (Cell Signaling, #3141), MLC (Cell Signaling, #3672), phospho-MLC (Cell Signaling, #3674), α-SMA (Abcam, ab5694), α-tubulin (Sigma-Aldrich, T5168), β-actin (Sigma-Aldrich, A5441) were used for immunoblotting or immunofluorescence on frozen sections. For *in vivo* experiments, Y27632 and PQ401 were obtained from AbMole Biosciences.

### Cell transfection

Genetic silencing of RhoA in MDA-MB-231 and CAF2 was performed by transducting cells with a lentivirus-vector-based shRNA (Sigma-Aldrich, clone number TRCN0000047711). A non-target shRNA sequence was used as a control vector (Sigma-Aldrich, SHC002). Enhanced green fluorescent protein (EGF)-expressing MDA-MB-231 cells were generated by transducing the cells with a pBMN-1-EGFP retroviral vector [56].

### *In vitro* collagen invasion assay

GFP^+^ expressing cancer cell spheroids (10^5^ cells/ml) alone or in presence of CAFs (5 × 10^4^ cells/ml) were seeded in a 3 mg/ml collagen I-DMEM gels (pH 7.2) and supplemented with 10% FCS. At this concentration, CAFs did not induce collagen gel contraction. In each experiment approximately 20 spheroids per condition were imaged with an inverted brightfield/epifluorescent microscope (Olympus IX70, PRIOR automated stage, OpenLab software) and 4× 1.00 lens at multiple time points for seven days. Analysis of invasion areas and cell scattering were performed with ImageJ software. Briefly, invasion area was measured as an ellipse formed by the most distal GFP^+^ cells from the spheroid center. The normalized invasion area was defined as the GFP ratio of the invasion area at a given time to size of the spheroid at d0. Cell scattering was defined as the number of GFP-positive single cells normalized to size of the spheroid at d1. The number of GFP-positive single cells was counted in automated fashion, after applying size- and intensity-thresholds to eliminate non-specific signal.

### Gene expression analysis

RNA was extracted using a RNeasy Mini Kit (Qiagen) and converted to cDNA using an iScript cDNA Synthesis Kit (Bio-Rad). The cDNA quality and concentration were measured with a ND-200 spectrophotometer (Nanodrop Technologies). Expression of EMT and motility-related genes was first determined by PCR array (Qiagen) and validated by RT-qPCR. RT-qPCR reaction was performed with Power SYBR Green Mix (Applied Biosystems). For human *IGF1* and *SERPINE1* detection, we used the following: *IGF1* forward primer, 5′-AAGGAGGCTGGAGATGTATTGC-3′; *IGF1* reverse primer, 5′-CGGACAGAGCGAGCTGACTT-3′; *SER-PINE1* forward primer, 5′-CACAAATCAGACGGCA GCACT-3′; *SERPINE1* reverse primer, 5′-CATCGGGCG TGGTGAACTC′-3′. The relative gene expression was analyzed with the 2^−ΔΔCt^ method.

### Immunoblotting

Total proteins were extracted using RIPA buffer supplemented with protease and phosphatase inhibitor mixtures (Roche). Denatured proteins were analyzed on a reducing SDS-polyacrylamide gel and blotted to a PVDF membrane by electrotransfer. Membranes were blocked for 1 h at room temperature, with 5% non-fat dry milk in TBST and incubated overnight at 4°C with primary antibodies. Protein detection was performed by using chemiluminescence with ECL or ECL+ reagents (GE healthcare). The relative intensity, shown as fold change over the control, was quantified with Image J software and represents the intensity of each protein band analyzed normalized to the intensity of either β-actin or GAPDH.

### RhoA activation assay

To determine RhoA activation in cancer cells co-cultured with CAFs, active RhoA-GTP was precipitated with Rhotekin-Rho-binding domain (RBD) glutathione affinity beads, using a RhoA Activation Assay Kit (Cytoskeleton) according to the manufacturer instructions. In tumor samples, RhoA activation was measured in tumor lysates with G-LISA RhoA Activation Assay Biochem Kit (Cytoskeleton).

### Detection of secreted IGF-1

CAF were cultured alone or in presence of cancer cells in serum-free medium. Supernatants were collected after 72 h and IGF-1 was measured by ELISA, using human IGF-1 Quantikine ELISA kit (R&D Systems) according to the manufacturer instructions. All IGF-1 values were normalized to amount of total proteins in the supernatant.

### *In vivo* tumor xenograft study

2 × 10^6^ MDA-MB-231 cells were implanted in the mammary fat pad of SCID mice (≥13 mice per group). Once tumors reached a tumor volume of approximately 40 mm^3^, mice were treated intraperitoneally with Y27632 [25 mg/kg], PQ401 [50 mg/kg] or Y27632 combined with PQ401, 3 times per week. Tumor volumes were measured every 2 to 3 days and volumes were based on the formula: $\frac{4}{3}\times \pi \times \frac{L}{2}\times {\left(\frac{l}{2}\right)}^{2}$. After 18 days, primary tumors were resected and processed for protein extraction or immunohistochemistry. Five weeks after primary tumor resection, mice were sacrificed and lung metastases were counted in whole lung sections stained with hematoxylin and eosin. Stained slides were quantified with Image J. The lung metastasis burden was defined as the fraction of image occupied by the metastases.

### Immunohistochemistry

Resected tumors were fixed in 4% paraformaldehyde, soaked in sucrose solution for 24 h and embedded in optimum cutting temperature compound (OCT) (Sakura Finetek). Frozen sections were cut into 20 μm sections, stained for α-SMA and phospho-ERM and mounted in Vectashield with DAPI counterstain (Vector Laboratories). Confocal fluorescence images were acquired with an Olympus BX61WI microscope and 20× water immersion lens (Fluoview software). The content of each protein analyzed was determined by measuring the number of pixels above a threshold value that was set based on the average intensity value of pixels from all slides under analysis.

### Statistical analysis

Data are expressed as mean ± SEM. Statistical analyses were carried out using Prism 6.0 Software. The statistical significance between groups in normally distributed continuous variables was determined using Student's *t*-test, one-way or two way ANOVA coupled with Bonferroni's or Dunnett's *post hoc* test. For non-Gaussian distributed variables, the statistical significance between groups was determined using Mann–Whitney test or Kruskal-Wallis test coupled with Dunn's *post hoc* test. Tests were considered significant when *P*-values were ≤ 0.05.

## SUPPLEMENTARY MATERIALS FIGURES


