# Supplementary material for: The crosstalk between breast carcinoma-associated fibroblasts and cancer cells promotes RhoA-dependent invasion via IGF-1 and PAI-1
Source: Oncotarget. 2017 Dec 28;9(12):10375–87. doi: 10.18632/oncotarget.23735 (PMC5828213; doi:10.18632/oncotarget.23735)
Supplement: Supplementary file 1 [file oncotarget-09-10375-s001.pdf]

The crosstalk between breast carcinoma-associated fibroblasts and cancer cells promotes RhoA-dependent invasion *via* IGF-1 and PAI-1

SUPPLEMENTARY MATERIALS

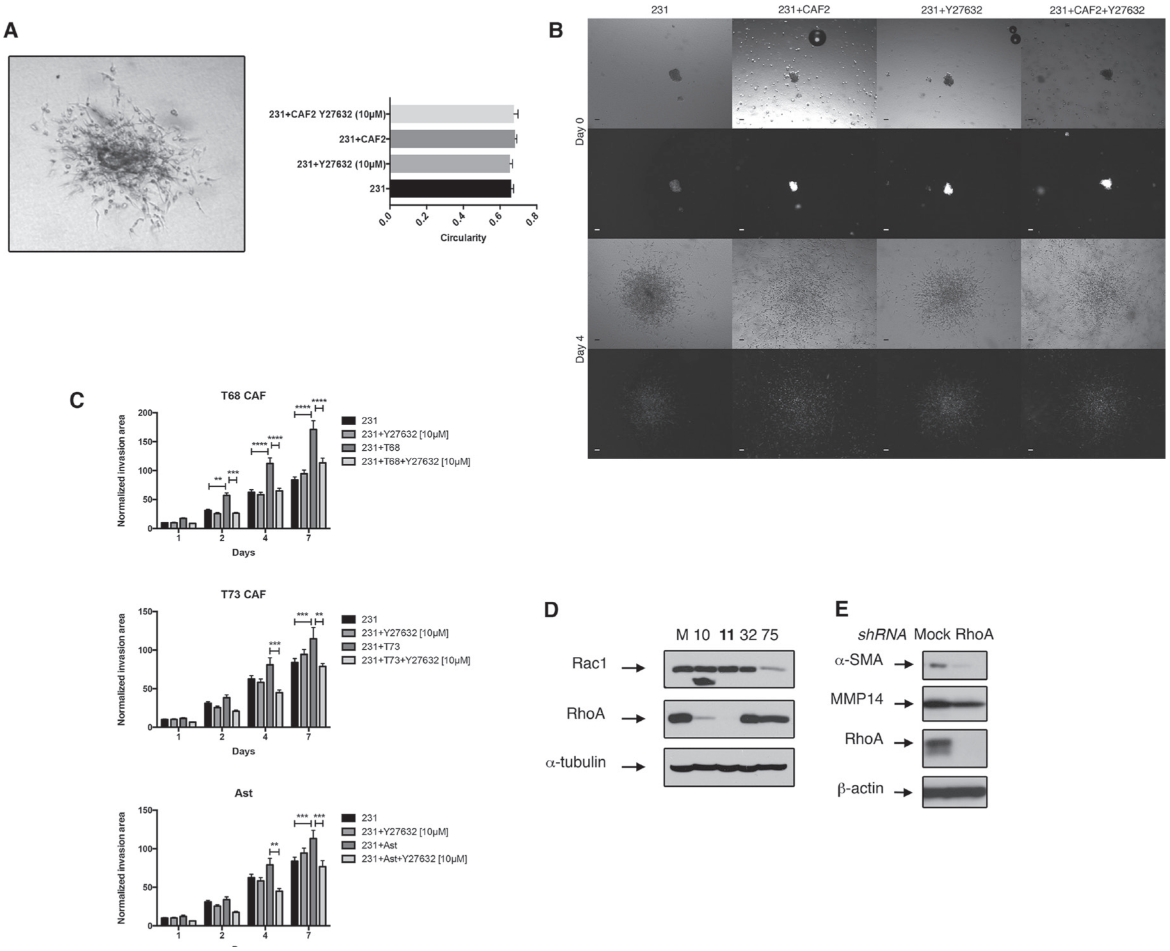

Supplementary Figure 1: ROCK inhibition reduces CAF-induced scattering and invasion in MDA-MB-231. (**A**) MDA-MB-231 spheroids cultured with CAF2 and imaged at day 1. (**B**) GFP⁺ MDA-MB-231 spheroids cultured without or with CAF2 in a collagen gel and treated with [10 μM] Y27632, imaged at day 4. (**C**) Kinetic of MDA-MB-231 cells invasion with or without TNBC CAFs (3) in collagen gels, and treated with [10 μM] Y27632 over a 7-day period. Data expressed as mean ± SEM. (**D**) Expression of RhoA (clones 10 and 11) and Rac1 (clones 32 and 75) in MDA-MB-231 cells genetically silenced by shRNA interference. Clone 11 was chosen for all subsequent experiments and compared to mock-transfected (M) cells. (**E**) Effect of RhoA silencing on α-SMA and MMP14 expression in CAF2: CAF2 genetically silenced by shRNA interference (clone 11) and assayed for α-SMA and MMP14 expression by immunoblotting β-actin was used as a loading control. Bar 100 μm. $^{**}p \leq 0.01$, $^{***}p \leq 0.001$, $^{****}p \leq 0.0001$.

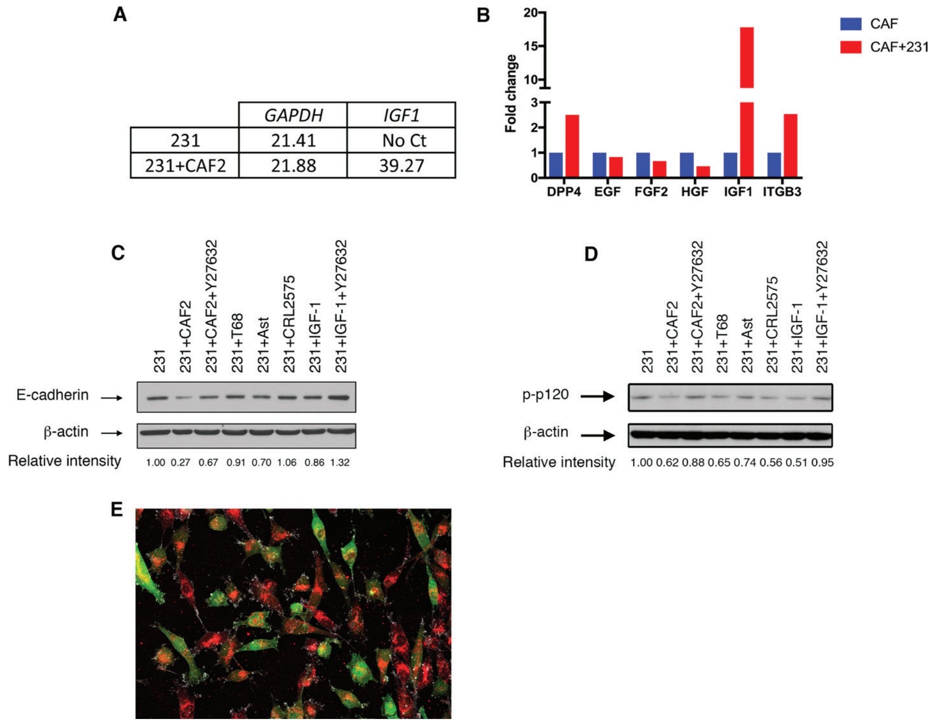

Supplementary Figure 2: Effect of the crosstalk between breast carcinoma-associated fibroblasts and cancer spheroids on cytokine and *adherens* junction protein expression. (**A**) MDA-MB-231 spheroids were culture alone or in presence of CAFs for 72 h and assayed for *IGF1* expression by RT-PCR. (**B**) CAFs were culture alone or in presence of MDA-MB-231 cells for 72 h and assayed for *DDP4, EGF, FGF2, HGF, IGF1* and *ITGB3* expression by RT-PCR array. (**C–D**) MDA-MB-231 cell spheroid were cultured alone or in presence of CAF2, IGF-1 [50 nM] and/or Y27632 [10 μM] for 72 h and immunoblotted for E-cadherin and phospho-p120 (Y228) catenin. Relative intensity, in fold change over MDA-MB-231 cell spheroid alone, represents the ratio of E-cadherin or phospho-p120 catenin to the loading control. (**E**) GFP^{+} MDA-MB-231 spheroids cultured in a collagen gel and immunostained for E-cadherin (red). Magnification: 60×.

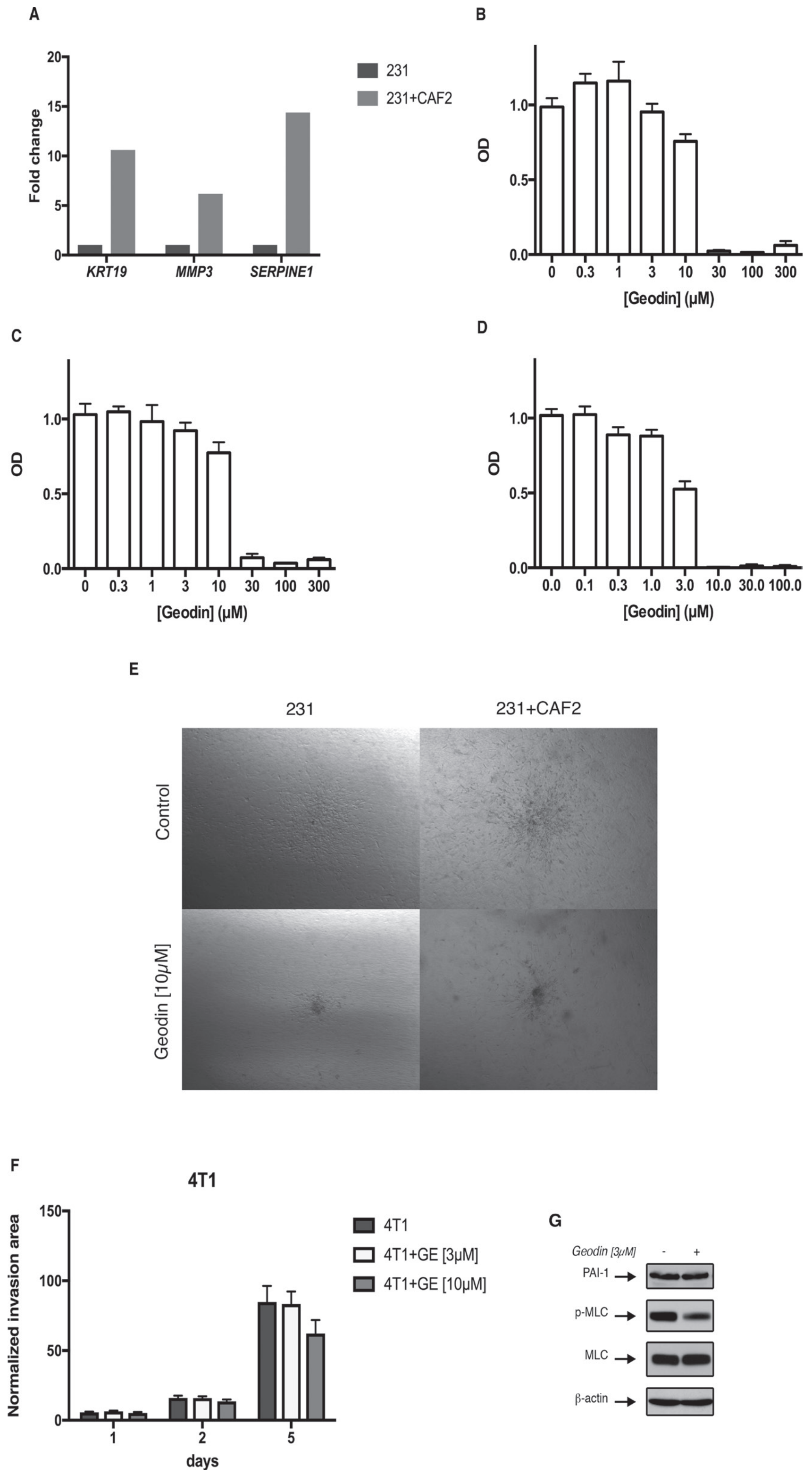

Supplementary Figure 3: Geodin reduces cell proliferation and decreases phospho-MLC expression in HUVECs. (**A**) MB-231 spheroids were culture alone or in presence of CAFs for 72 h and assayed for *KRT19, MMP3* and *SERPINE1* expression by RT-PCR array. (**B**) MDA-MB-231 cells, (**C**) CAF2 and (**D**) HUVECs were treated with increasing doses of geodin for 72 h and assayed for cell proliferation by MTT assay. (**E**) Geodin inhibits the invasion of 231 cells cultured with or without CAFs. (**F**) Effect of geodin on collagen invasion of 4T1 cells. Geodin has no effect on the invasion of 4T1 cells. (**G**) HUVECs were treated with 3 µm of geodin for 72 h and assayed for phospho-MLC (Thr18/Ser19), MLC and PAI-1 expression by immunoblotting. Geodin decreases phospho-MLC without affecting PAI-1 expression.

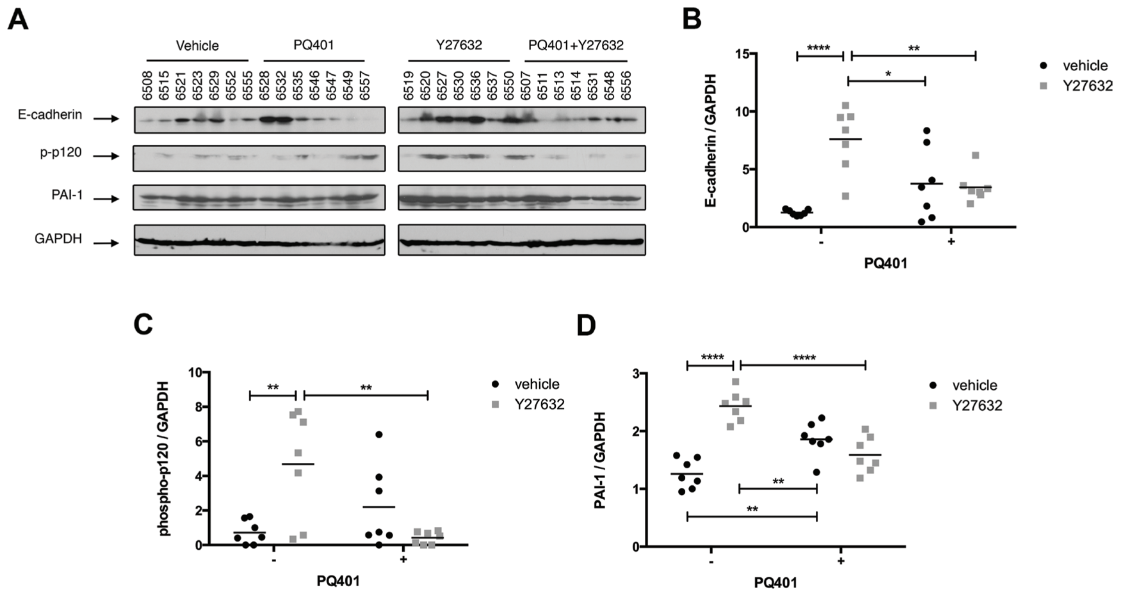

Supplementary Figure 4: ROCK inhibition increases *adherens* junction expression in breast cancer xenografts. MDA-MB-231 cells were implanted in the mammary fat pad of SCID mice and treated with PQ401, Y27632 or PQ401 combined to Y27632. After 18 days of treatment, tumors were harvested and assayed for E-cadherin, phospho-p120 catenin and PAI-1 expression by immunobloting**.**

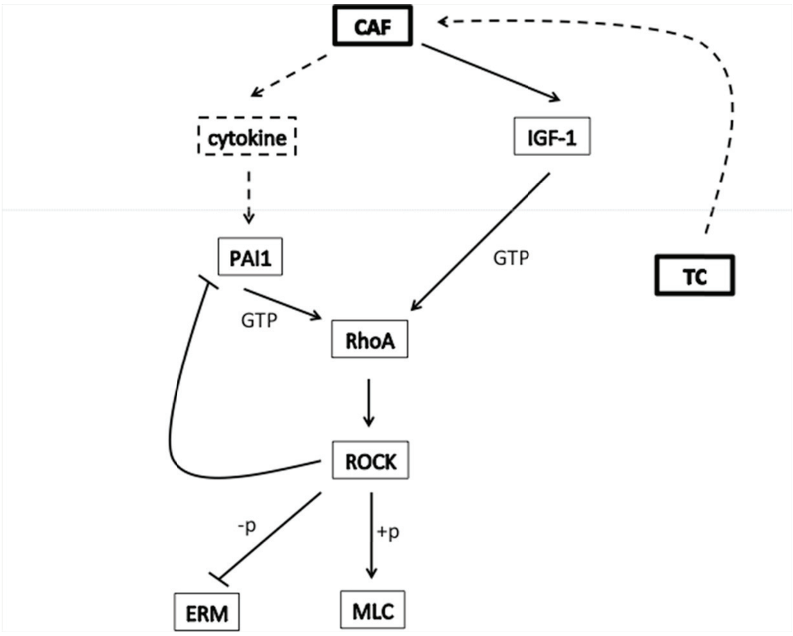

Supplementary Figure 5: Summary of the CAF-cancer cell crosstalk. In presence of CAFs, RhoA signaling pathway (RhoA/ROCK/MLC) is activated in cancer cells *via* IGF-1 secretion and PAI-1 up-regulation. Blockade of ROCK with Y27632 leads to MLC phosphorylation, as well as to ERM phosphorylation and PAI-1 up-regulation (regulatory loop).

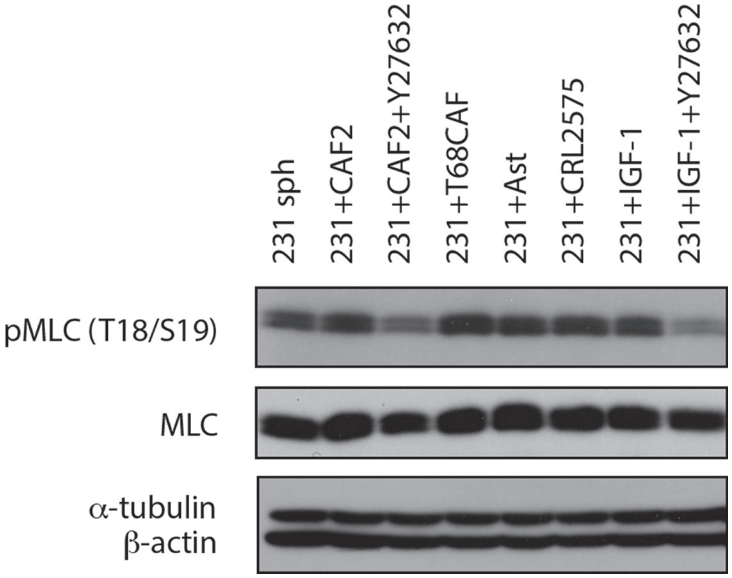

Supplementary Figure 6: Y27632 decreases phospho-MLC in MDA-MB-231 co-cultured with CAF2. MDA-MB-231 cell spheroids were cultured alone or in presence of CAFs, IGF-1 [50 nM] and/or Y27632 [10 µM] for 72 h and immunoblotted for MLC, phospho-MLC (Thr18/Ser19) and β-actin as loading control**.**

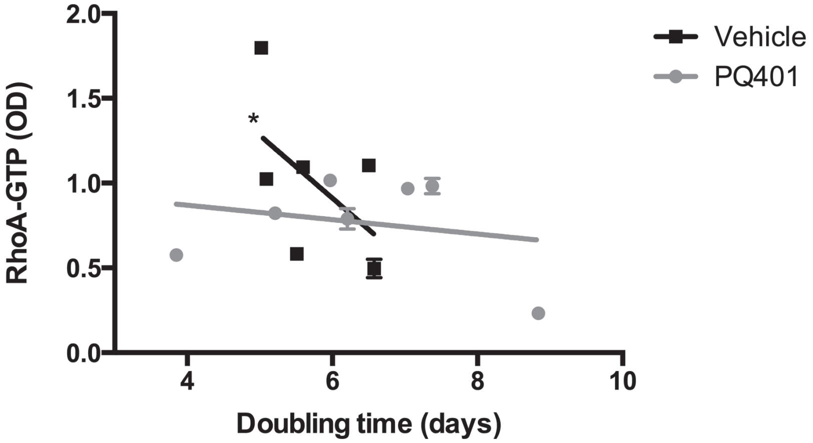

Supplementary Figure 7: High RhoA-GTP expression is associated with shorter tumor volume doubling time in MDA-MB-231 xenograft. The control group (vehicle) but not PQ401-treated shows a significant negative correlation between RhoA-GTP expression and tumor volume doubling time.

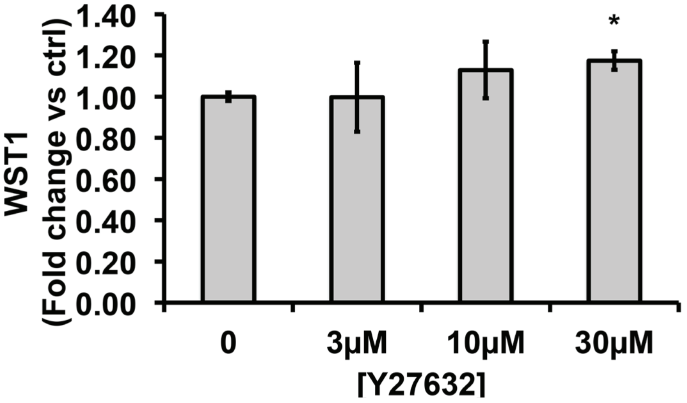

Supplementary Figure 8: Y27632 increases survival of MDA-MB-231 spheroids. MDA-MB-231 were cultured on low-adhesion surface for 24 h and then treated with Y27632 [3–30 μM] for 72 h. Cell viability was measured by WST1 assay. $^{*}p \leq 0.05$.
